# Domestic

**Published:** 1841-03-13

**Authors:** 


### DOMESTIC.

### HEALTH OF THE CITY.

Interments *in the City and Liberties of Philadelphia, from the 27th of February to the 6th of March*, 1841.

| Diseases. | Adults. | Children. | Diseases. | Adults. | |
|---|---|---|---|---|---|
| Apoplexy, | 1 | 0 | *Brought forward,* | 33 | 55 |
| Burns, | 2 | 0 | Measles, | 0 | 5 |
| Casualty, | 0 | 1 | Old age, | 1 | 0 |
| Croup, | 0 | 4 | Œdema of lungs, | 1 | 0 |
| Congestion, | 0 | 1 | Palsy, | 1 | 0 |
| —— of brain, | 0 | 1 | Rupture of a blood- | | |
| Consumption of | | | vessel, | 1 | 0 |
| the lungs, | 10 | 1 | Strangulation, | 1 | 0 |
| Convulsions, | 2 | 4 | Small pox, | 1 | 3 |
| Dropsy of head, | 0 | 5 | Still-born, | 0 | 8 |
| Dis'se of the brain, | 0 | 1 | Spitting of blood, | 0 | 1 |
| —— heart, | 1 | 0 | Unknown, | 0 | 1 |
| —— lungs, | 0 | 2 | | — | — |
| Dysentery, | 1 | 0 | Total, 112— | 39 | 73 |
| Debility, | 1 | 4 | —— | | |
| Epilepsy, | 1 | 1 | Of the above, there | | |
| Fever, typhus, | 2 | 0 | were under 1 year | | 39 |
| —— typhoid, | 2 | 0 | From 1 to 2 | | 15 |
| —— puerperal, | 1 | 0 | 2 to 5 | | 9 |
| —— scarlet, | 0 | 2 | 5 to 10 | | 6 |
| Inflammation of | | | 10 to 15 | | 3 |
| the brain, | 0 | 3 | 15 to 20 | | 0 |
| —— bronchi, | 2 | 8 | 20 to 30 | | 12 |
| —— lungs, | 4 | 10 | 30 to 40 | | 8 |
| —— breast, | 0 | 2 | 40 to 50 | | 5 |
| —— heart, | 1 | 0 | 50 to 60 | | 8 |
| —— peritonæum, | 1 | 0 | 60 to 70 | | 4 |
| Influenza, | 1 | 0 | 70 to 80 | | 1 |
| Inanition, | 0 | 2 | 80 to 90 | | 2 |
| Marasmus, | 0 | 2 | 90 to 100 | | 0 |
| | — | — | | | —— |
| *Carried forward,* | 33 | 55 | Total, | | 112 |

In the above are included 23 people of colour, 4 interments from the alms-house, and 1 from the country.

